# The Moderating Effect of Social Capital on the Relationship Between Loneliness and Depression in Older Adults

**DOI:** 10.3390/bs15091157

**Published:** 2025-08-25

**Authors:** Eun Seo Jeong, Sung Man Bae

**Affiliations:** 1 Department of Psychology, Graduate School, Dankook University, Cheonan 31116, Republic of Korea; agtj47@naver.com; 2 Department of Psychology and Psychotherapy, Dankook University, Cheonan 31116, Republic of Korea

**Keywords:** older adults, loneliness, depression, social capital, structural social capital, cognitive social capital

## Abstract

Loneliness contributes to depression in older adults, but research on effective intervention strategies remains limited. This study aims to examine the moderating effects of social capital on the relationship between loneliness and depression in older adults. Social capital was divided into structural and cognitive social categories. A self-report questionnaire was administered to 350 participants aged 65 or older, using the Korean version of the UCLA Loneliness Scale, Short-Form Geriatric Depression Scale, Structural Social Capital Scale, and Cognitive Social Capital Scale. Data from 334 patients were included in the final analysis, and the moderating effect was tested using Model 1 of the PROCESS Macro for SPSS. The results of this study are as follows: (1) it demonstrated that loneliness is a significant predictor of higher levels of depression; (2) structural social capital significantly moderated this relationship, indicating that the impact of loneliness on depressive symptoms varied according to the level of structural social capital; (3) cognitive social capital also moderated this relationship, suggesting that the influence of loneliness on depression differs based on the level of cognitive social capital. This study provides valuable foundational data for the development of prevention and intervention strategies targeting loneliness and depression in old age.

## 1. Introduction

The increase in the population of older adults and rising life expectancy is gradually changing their lives and roles. Their physical and mental health, as well as successful aging, have emerged as important social issues (United Nations, 2023). Old age is often accompanied by poverty due to retirement, physical decline caused by aging, chronic pain, cognitive deterioration, and a profound sense of loss, which collectively heighten the risk of depression and suicide. In particular, depression in older adults can contribute to the onset or worsening of chronic diseases, decline in cognitive function, dementia, pain, and physical health issues (Alexopoulos, 2005; Kee, 1999; Diniz et al., 2013). It also increases the risk of impairments in activities of daily living and mobility (Penninx et al., 1999).

Loneliness is a key risk factor of depression in older adults (S. L. Lee et al., 2021). It is a distressing emotional state characterized by feelings of emptiness that occurs when there is a gap between an individual’s expectations and actual experiences in social relationships (Seo et al., 2020). Loneliness and social isolation are distinct concepts, and loneliness can be experienced even without being isolated (Perlman & Peplau, 1981; Jin & Bae, 2023).

S. L. Lee et al. (2021) reported that the association between loneliness and depression remained significant, even after 12 years, after controlling for social isolation, social support, and genetic risk factors in a large sample of adults over the age of 50. The greater the loneliness, the more depressive symptoms were reported; this effect was particularly pronounced in older adults (Oliveira et al., 2019). Chronic experiences of loneliness have been shown to overactivate the hypothalamic–pituitary–adrenal axis, resulting in elevated cortisol levels, autonomic dysregulation, increased heart rate, and heightened inflammatory responses (Brown et al., 2018). This can contribute to the onset of various diseases, and the deterioration of physical health leads to difficulties in maintaining daily functioning and a loss of independence, thereby increasing the risk of depression (Vaughan et al., 2016; Ormel et al., 2002). Additionally, individuals who feel lonely can form negative perceptions of social interactions and negative cognitive schemas, which can increase their risk of depression (Hawkley & Cacioppo, 2010).

In old age, according to Socioemotional Selectivity Theory, older adults perceive their remaining time as limited and thus prioritize emotionally meaningful decisions, leading them to reduce the size of their social networks in order to increase the emotional closeness within those networks (Lang & Carstensen, 2002). Additionally, the size of the social network decreases due to the lack of social engagement, and various aspects of overall functioning simultaneously deteriorate (K. H. Kim et al., 2015; Ahmad, 2020). Older adults can strengthen their self-resilience and achieve successful aging through emotional, instrumental, and informational support from neighbors and friends (G. A. Oh & Lee, 2022).

The lack of trust, small social networks, limited social participation, and low normative awareness result in extensive dysfunction in terms of health, physical environment, social relationships, and psychological well-being (M. H. Lee & Ko, 2015). In summary, older adults can enhance their quality of life and contribute to healthy aging by reducing negative emotions through emotional support, social integration, and the exchange of information within social relationships.

Social capital refers to the sum of the actual and potential resources acquired through mutual understanding and interpersonal relationships. It is a form of capital that arises from social networks and interactions and is conceptually distinct from economic, human, and psychological capital (Bourdieu, 1986). That is, social capital is acquired not only by belonging to a network but also through ongoing interactions and relationships within that network (Coleman, 1988). In old age, social capital can be a useful resource to meet the need for social recognition while maintaining social participation and activities by forming continuous social relationships. According to Choi (2008), depression decreased when people actively participated in social activities with a high sense of community or solidarity, even after controlling for demographic variables (gender, age, education level, income, marital status, and health status). An et al. (2019) found that social capital significantly reduced depression by buffering the negative effects of stress, even after controlling for age, gender, marital status, and education level. While social capital can be divided into various types, it is generally categorized as cognitive and structural social capital. Structural social capital refers to relatively objective and externally observable elements, such as networks and social organizations (Grootaert & Bastelaer, 2002). Cognitive social capital refers to subjective and invisible elements, such as trust, reciprocity, norms, and values recognized in social relationships (Grootaert & Bastelaer, 2002). Harpham et al. (2002) argue that structural elements refer to the degree or scope of connection and intensity of activities in an organization, whereas cognitive elements include support, trust, reciprocity, and sharing. Thus, the current study included networks and social participation as structural social capital and trust and social support as cognitive social capital.

Cognitive social capital is associated with reduced levels of depression (Yun & Bae, 2020; Fujiwara & Kawachi, 2008), whereas the evidence regarding such effects of structural social capital remains unclear. According to Forsman et al. (2012), depression among older adults has a strong relationship with low structural social capital (frequency of social contact), and cognitive social capital (trust) showed a significant relationship. Fujiwara and Kawachi (2008) found that cognitive social capital (trust and sense of belonging) significantly reduces the risk of developing depression over a 2–3 year follow-up period, whereas structural social capital (volunteering and community participation) showed no significant association with depression.

Regarding empirical evidence for the moderating effect of social capital between loneliness and depression, Son et al. (2022) reported that spousal support moderated the effect of loneliness on depression in adults aged 65 or older. According to Goodfellow et al. (2022), social support and the frequency of conversations with neighbors moderated the relationship between loneliness and well-being. Shin and Park (2023) reported that both types of networks, characterized by various relationships, such as family and friends and family-centered network types, moderated the relationship between loneliness and depression. In an experimental study by Coll-Planas et al. (2017), interventions targeting the formation of networks between primary healthcare centers, senior centers, and other community assets in the neighborhood, along with social support and social participation interventions, led to decreases in loneliness and depressive symptoms among older adults.

Multiple studies have confirmed the influence of social capital on depression in old age but have limited its scope to family and friends; social capital must be expanded to include neighbors and colleagues (Shin & Park, 2023; S. J. Kim & Lee, 2022; K. H. Kim et al., 2015). In addition, the cognitive and structural categories of social capital should be regarded as distinct resources due to their differing roles; however, there is a lack of research that systematically classifies and integrates these components.

Among the sociodemographic variables, gender, age, economic level, and the presence of chronic diseases were confirmed to affect depression in older adults. Women had higher levels of depression than men (Sonnenberg et al., 2000; Jun, 2014), and the risk of depression increased with age (Blazer et al., 1991; Kwon, 2015). Depression increased with rising economic difficulties (Butterworth et al., 2009; T. W. Kim et al., 2015), and the risk of depression increased with the severity of chronic diseases (Huang et al., 2010).

In line with the prior literature, this study aims to examine the effect of loneliness on depression in older adults and explores the moderating roles of structural and cognitive social capital in this relationship. The specific hypotheses are as follows: first, as the level of loneliness in older adults increases, depression correspondingly rises. Second, structural social capital has a significant moderating effect on the relationship between loneliness and depression. Third, cognitive social capital significantly moderates the relationship between loneliness and depression.

## 2. Materials and Methods

### 2.1. Participants

This study conducted an online and offline survey targeting 350 adults aged 65 or older. The online survey used a Google survey form; the offline survey was conducted one-on-one, either self-administered or with the researcher reading the questions aloud, depending on the participants who were willing to participate after reading the recruitment notice. The survey consisted of 87 questions and took approximately 30 min to complete. All participants consented to the research participation guide before the survey and were informed that they could stop participating at any point. In the case of the online survey, additional information was provided that as the survey was collected anonymously, it could not be withdrawn after submission. Participants who completed the survey were compensated with a beverage gift certificate or snack. Of the 350 participants, 16 were excluded for not meeting the age criterion (under 65 years) or having incomplete survey responses; a final sample of 334 participants was used for analysis. Of the 334 participants, 22 completed the offline survey and 312 completed the online survey. There were 152 males (45.5%) and 182 females (54.5%) with an average age of 68.85 years (SD = 3.99). All research procedures in this study were approved by the Institutional Review Board of Dankook University (Approval Number: 2024-05-017-004).

### 2.2. Measurement Tools

#### 2.2.1. UCLA Loneliness Scale

To measure loneliness, the revised UCLA Loneliness Scale developed by Russell et al. (1980), translated into Korean by K. H. Kim and Kim (1989), was used. It consists of 20 items (10 positive and 10 negative), and responses are recorded on a 4-point Likert scale (1 = “not at all” to 4 = “often”). The positive items (1, 4, 5, 6, 9, 10, 15, 16, 19, and 20) are reverse scored. Higher scores indicate higher levels of loneliness. Sample items on the scale include “I get along well with people around me,” “I feel a sense of belonging to the group,” and “My interpersonal relationships are superficial.” In a study by K. H. Kim and Kim (1989), Cronbach’s α was 0.84, and in this study, it was 0.95.

#### 2.2.2. Short-Form Geriatric Depression Scale

To measure geriatric depression, we used the Short-Form Geriatric Depression Scale (SGDS-K), which was validated in Korean by Cho et al. (1999) and based on the SGDS scale simplified by Yesavage et al. (1982). This scale consists of 15 items (5 positive and 10 negative), regarding the participants’ mood during a week. Positive items 1, 5, 7, 11, and 13 are reverse scored. All items are answered with a yes/no; a higher score indicates higher levels of depression. The items include “Are you generally satisfied with your current life?,” “Have you recently lost a lot of activity or motivation?,” and “Do you feel like a useless person now?” In the study by Cho et al. (1999), Cronbach’s α was 0.91, and in this study, it was 0.91.

#### 2.2.3. Structural Social Capital Scale

To measure social networks, the Korean version of the Lubben Social Network Scale (LSNS-18), validated by Y. S. Kim (2020), consisting of 18 items, was used. To measure social participation, the Korean version of the World Health Organization (WHOQOL–BEEF) standardized scale, revised and supplemented by M. A. Kim (2018) with 8 items for the structural social capital scale, was used. The LSNS-18 is used to measure the social networks of relatives, friends, and neighbors of older adults and to screen older adults in social isolation situations. It consists of six questions each on family networks, friends, and neighbors. A higher score indicates a larger social network size. The questions include “How many relatives do you see or contact at least once a month?,” “How often do you meet or contact your most frequent friends?,” and “How many neighbors do you feel comfortable talking about personal matters with?” In Y. S. Kim’s (2020) study, Cronbach’s α was 0.89, and in this study, it was 0.94. The social participation scale was divided into economic activities, volunteer activities, social–friendship activities, and self-development activities. The amount of activity and participation in each area was evaluated using a 5-point Likert scale (1 = “0 times/0 h” to 5 = “7 times or more/4 h or more”). A higher score indicates a higher level of social participation. Examples of items include “How many times a week do I engage in economic activities?” and “How many hours do I spend on social activities?” In this study, Cronbach’s α was 0.70. The Cronbach’s α of structural social capital was 0.93.

#### 2.2.4. Cognitive Social Capital Scale

To measure cognitive social capital, seven items related to trust were extracted from the questionnaire developed by Y. H. Lee et al. (2006) and were adapted to the current study. Additionally, eight items were used to measure social support, drawn from the original PSO scale (Perceived Social Support through Others Scale) developed by Park (1985) and the abbreviated PSO-8 version revised by H. R. Kim et al. (2021). The trust scale measures the degree of trust in personal and social objects, and the items include “I trust my family,” “I trust my friends,” and “I trust my neighbors”. In this study, Cronbach’s α was 0.82. The social support scale (PSO-8) assesses the degree of social support received daily. The items include “They make me feel needed and valuable to them” and “They make me feel proud of what I do.” In the study by H. R. Kim et al. (2021), Cronbach’s α was 0.95, and in this study, it was 0.94. Responses are received on a 5-point Likert scale (1 = “not at all” to 5 = “very much”), and a higher score indicates higher cognitive social capital. In this study, Cronbach’s α of cognitive social capital was 0.95.

### 2.3. Data Analysis

The data collected in this study were analyzed using IBM SPSS Statistics version 23.0 and Hayes’s (2012) PROCESS Macro for SPSS version 4.2. First, a frequency analysis was conducted, using SPSS 23.0, to identify demographic characteristics. Second, descriptive statistical analysis was conducted to confirm the mean, standard deviation, skewness, and kurtosis of the major variables. Additionally, Pearson’s correlation analysis was performed to assess the relationship between major variables. Third, PROCESS Macro Model 1 was used to verify the moderating effects of structural and cognitive social capital on the relationship between loneliness and depression (Hayes, 2012). To minimize the possibility of multicollinearity, mean centering was applied to analyze the interaction effects. Finally, based on the suggestion of Frazier et al. (2004), the significance of the simple regression line between the independent and dependent variables was verified at specific values (mean − 1SD, mean, mean + 1SD) of the moderator variables.

## 3. Results

### 3.1. Demographic Characteristics

Out of a total of 334 respondents, 152 (45.5%) were male and 182 (54.5%) were female, with an average age of 68.85 years (SD = 3.99). Furthermore, 226 (67.7%) were aged 65–69, 85 (25.4%) were 70–74, 11 (3.3%) were 75–79, and 12 (3.6%) were 80–84. The most common marital status was married (225, 76.3%), followed by widowed (43, 12.9%), single (25, 7.5%), and divorced (11, 3.3%). The most common type of living arrangement was with a spouse (207, 62.0%), followed by with children (27, 8.1%), with a spouse and children (35, 10.5%), alone (64, 19.2%), and others (1, 0.3%). The subjective health status was “good” for 12 (3.6%), “Fairly-good” for 60 (18.0%), “Fair” for 182 (54.45%), “Fairly-poor” for 65 (19.5%), and “poor” for 15 (4.5%) respondents. Chronic diseases included diabetes in 125 (37.4%), hypertension in 112 (33.5%), stroke in 27 (8.1%), arthritis in 58 (17.4%), others in 3 (0.9%), and none in 102 (30.5%) respondents. The specific demographic variables are presented in Table 1.

### 3.2. Correlation

Before hypothesis testing, descriptive statistics, including the mean and standard deviation of the major variables and the correlation between the variables, were calculated, as shown in Table 2. Loneliness showed a significant positive correlation with depression (r = 0.784, *p* < 0.001). Significant negative correlations were found between loneliness and structural social capital (r = −0.640, *p* < 0.001) as well as between loneliness and cognitive social capital (r = −0.805, *p* < 0.001). Structural social capital (r = −0.621, *p* < 0.001) and cognitive social capital (r = −0.707, *p* < 0.001) both showed significant negative correlations with depression. Structural and cognitive social capital (r = 0.683, *p* < 0.001) showed a significant positive correlation.

### 3.3. Moderating Effect of Structural Social Capital on the Relationship Between Loneliness and Depression

To verify the effect of different levels of structural social capital on the relationship between loneliness and depression, PROCESS Macro Model 1 was utilized (Table 3; Hayes, 2012). Gender had a negative effect on depression (B = −0.838, t = −2.604, *p* < 0.01), but the presence of chronic diseases (B = 0.919, t = 2.705, *p* < 0.01) had a positive effect on depression. Loneliness had a positive effect on depression, even after controlling for covariates (B = 0.199, t = 12.901, *p* < 0.001), whereas structural social capital (B = −0.049, t = −4.485, *p* < 0.001) had a negative effect on depression. In addition, the interaction term between loneliness and structural social capital (B = −0.002, t = −3.350, *p* < 0.001) also showed a significant effect on depression. Hence, the effect of loneliness on depression varies according to the level of structural social capital. This indicates that higher levels of loneliness are associated with increased depression, whereas higher structural social capital mitigates the effects of loneliness on depression.

To verify the statistical significance of this interaction effect, the significance of the simple regression line between loneliness and depression was tested at specific values (mean − 1SD, mean, mean + 1SD) of the moderating variable, structural social capital (Table 4). All simple regression lines of loneliness on depression were significant, and the group with low structural social capital (B = 0.230, t = 14.638, *p* < 0.001) showed a stronger positive relationship between loneliness and depression than the group with high structural social capital (B = 0.168, t = 8.415, *p* < 0.001).

### 3.4. Moderating Effect of Cognitive Social Capital on the Relationship Between Loneliness and Depression

To verify whether the relationship between loneliness and depression in older adults varies according to the level of cognitive social capital, PROCESS Macro Model 1 was used (Table 5; Hayes, 2012).

Gender had a negative effect on depression (B = −0.673, t = −2.024, *p* < 0.05), and the presence of chronic diseases had a positive effect on depression (B = 0.625, t = 2.671, *p* < 0.01). Loneliness had a positive effect on depression, even after controlling for covariates (B = 0.201, t = 10.530, *p* < 0.001), whereas cognitive social capital had a negative effect on depression (B = −0.066, t = −3.042, *p* < 0.01). In addition, the interaction term between loneliness and cognitive social capital had a significant effect on depression (B = −0.002, t = −2.763, *p* < 0.01). Thus, the effect of loneliness on depression may vary depending on the level of cognitive social capital.

To verify the statistical significance of this interaction effect, the significance of the simple regression line between loneliness and depression was tested at specific values (mean − 1SD, mean, mean + 1SD) of the moderating variable, cognitive social capital (Table 6). All simple regression lines of loneliness on depression were significant, and the group with low cognitive social capital (B = 0.226, t = 10.950, *p* < 0.001) showed a stronger positive relationship between loneliness and depression than the group with high cognitive social capital (B = 0.176, t = 8.158, *p* < 0.001).

## 4. Discussion

This study aimed to examine whether structural and cognitive social capital moderated the relationship between loneliness and depression in adults aged 65 and older. The discussion and implications of this study are as follows.

First, a significant positive association was observed between loneliness and depression among older adults. These results are consistent with those of previous studies, confirming that loneliness is a major predictor of depression (S. L. Lee et al., 2021; Hawkley & Cacioppo, 2010; Pak & Bae, 2025; Hsueh et al., 2019). According to Lieberz et al. (2021), groups with high levels of loneliness exhibit decreased trust and emotional responses to positive conversations. Hutten et al. (2021) found that lonely individuals tend to perceive less social support by negatively distorting their social environment. In other words, feeling lonely can cause negative distortions regarding social environment, further decreasing trust and perceptions of support, thereby increasing depression.

Second, structural social capital demonstrated a significant negative relationship with depression. Previous studies have reported mixed results, with some showing no link between structural social capital and depression and others indicating that it increases depression (Wu et al., 2016; Bassett & Moore, 2013). Older adults can secure resources to cope with depression through social networks (M. I. Kim et al., 2019). As contact with other people increases, vitality and positive emotions increase, which may reduce depression (Choi, 2008; R. Wang et al., 2020). Additionally, the structural elements of social capital promote the functioning of cognitive elements (Grootaert & Bastelaer, 2002). Structural social capital provides opportunities for older adults to form cognitive social capital, interact with others, and build trust based on these interactions (Lu & Peng, 2019; Nilsson & Mattes, 2015). Through social participation activities, older adults gain trust and a sense of belonging in the community; individuals who trust their neighbors are more likely to participate in various social activities in the community (Bae, 2021; Lu & Peng, 2019).

Third, structural social capital significantly moderated the relationship between loneliness and depression. Higher levels of structural social capital lessen the influence of loneliness on depression, suggesting it may serve as a protective factor. This result is consistent with the study by Domènech-Abella et al. (2017), which suggests that the growing size of the social network may reduce depression in a group of lonely people. It is similar to a study by Zhao and Wu (2022), affirming that social participation can reduce loneliness. Social loneliness arises from the perception of a lack of social networks with shared common interests or activities (Weiss, 1973). Thus, participation in social activities can help reduce loneliness and alleviate depression by facilitating the formation of relationships with others sharing similar interests (Levasseur et al., 2017; Burroughs & Wilkie, 2016). The larger the social network size, the more benefits of the social capital, such as trust and emotional and instrumental support (Seeman & Berkman, 1988). Therefore, social network expansion can promote social interaction and activate social participation, thereby preventing social isolation and loneliness from developing into depression (Domènech-Abella et al., 2017).

Fourth, cognitive social capital demonstrated a significant negative relationship with depression. This finding is consistent with that of S.-S. Kim et al. (2012), who found that the onset and chronic course of depression were more frequent in the group with low interpersonal trust than the group with high interpersonal trust. Grav et al. (2012) found that the risk of depression increased as emotional and instrumental support decreased. Social support helps people solve problems and escape from stressful situations by enhancing their emotional stability (Seo & Lee, 2014). High levels of trust foster a positive psychological state, such as “being accepted” within a community, which helps prevent depressive symptoms (Fujiwara & Kawachi, 2008).

Fifth, cognitive social capital significantly moderated the relationship between loneliness and depression. In other words, the magnitude of the influence of loneliness on depression varied depending on the level of cognitive social capital. This result is consistent with those of previous studies, showing that perception of abundant support resources reduces depression, even with high levels of loneliness. Emotional loneliness occurs in the absence of deep mutual bonds in relationships such as close family, friends, and lovers (Weiss, 1973). However, cognitive social capital can alleviate emotional loneliness by increasing trust, social support, and mutual expectations that individuals have toward others or the community (Forsman et al., 2011). Social support allows people to feel cared for and valued (Cobb, 1976), and this trust forms relationships that can be relied upon in difficult and important moments (Rempel et al., 1985). Thus, trust and social support can promote deep mutual bonds, thereby reducing loneliness and subsequently alleviating depressive symptoms (Forsman et al., 2011).

The contributions and implications of this study are as follows: First, it significantly expands existing research by verifying the moderating effects of structural and cognitive social capital on the relationship between loneliness and depression. The results of this study suggest that forming structural capital through programs that expand social networks and promote participation in social activities can help lower the level of depression. In addition, efforts to increase cognitive social capital, through social skills training that fosters trust in others and facilitates emotional support, as well as interventions aimed at enhancing positive emotions, can lower levels of depression. Second, from a psychological perspective, there is no universal definition of social capital. This study categorized social capital into structural social capital and cognitive social capital and verified its moderating effect on depression by evaluating social network, social participation, social support, and trust as major components, thereby providing a conceptual framework of social capital from a psychological perspective. Third, as loneliness and social isolation were previously considered the same concept, intervention strategies to reduce loneliness have mainly focused on increasing social contact (structural social capital). Such intervention strategies do not sufficiently reflect the emotional aspects of loneliness. In this study, we aimed to consider interventions for the emotional and social aspects of loneliness by examining the structural (quantitative) and cognitive (qualitative) dimensions of social capital. Finally, we have added to evidence regarding the relationship between structural social capital and depression, considering the inconsistent findings of previous studies. Previous studies on the relationship between structural social capital and depression have yielded inconsistent results, consequently only highlighting the importance of cognitive social capital. This study clarified the role of structural social capital by confirming its direct effect on depression and its moderating effect on the relationship between loneliness and depression.

## 5. Conclusions

The limitations of this study and implications for future research are as follows: First, this study analyzed loneliness as a single dimension, highlighting the current lack of research on the relationship between the subfactors of loneliness and depression. Therefore, it is necessary to measure the multidimensional aspects of loneliness and expand on the relationship between loneliness and depression. Second, this study focused on social capital in face-to-face social relationships. Recently, the role of digital media in old age has received increasing attention owing to the acceleration of digital transformation (Bae, 2022; E. A. Oh & Bae, 2024). Online social capital can expand offline relationships and facilitate information exchange (Yoon et al., 2016; T. J. Wang, 2023). Therefore, further research is required to confirm the role of social capital in online relationships. Third, in this study, gender and chronic illness were included as covariates in the moderation model and significantly predicted depression. Prior research indicates that the impact of social capital on depression in older adults may vary by gender (Zhou et al., 2022). Moreover, when physical function declines due to chronic disease, physical limitations can lead to reduced social participation, which can exacerbate loneliness and lead to depression (Griffith et al., 2017). Therefore, future studies should examine more closely how gender and chronic illness may differentially shape the relationships among loneliness, social capital, and depression. Fourth, this study conducted a cross-sectional analysis; thus, limitations exist in the clear identification of the causal relationships between variables. As social capital is affected by accessibility and consistency over time, temporal factors should also be considered. Therefore, it is necessary to validate these results through future longitudinal studies.

## Figures and Tables

**Table 1:** Demographic characteristics (N = 334).

Variable		N	%
Gender	Male	152	45.5
	Female	182	54.5
Age	Mean (SD)	68.85 (3.99)	
Education	No formal education (illiterate)	4	1.2
	Primary school	37	11.1
	Middle school	93	27.8
	High school	147	44.0
	>High school	53	15.9
Marital Status	Married	255	76.3
	Single	25	7.5
	Divorced	11	3.3
	Widowed	43	12.9
Type of Living Arrangement	With spouse	207	62.0
	With children	27	8.1
	With spouse and children	35	10.5
	Living alone	64	19.2
	Others	1	0.3
Subjective Economic Status	Low	19	5.7
	Lower-middle	66	19.8
	Middle	202	60.5
	Upper-middle	42	12.6
	High	5	1.5
Perceived Health Status	Poor	15	4.5
	Fairly poor	65	19.5
	Fair	182	54.5
	Fairly good	60	18.0
	Good	12	3.6
Chronic Diseases	None	102	30.5
	Diabetes	125	37.4
	Hypertension	112	33.5
	Stroke	27	8.1
	Arthritis	58	17.4
	Others	3	0.9

**Table 2:** Correlations, means, standard deviation (N = 334).

	1	2	3	4
1. Loneliness	-			
2. Structural Social Capital	−0.640 ***	-		
3. Cognitive Social Capital	−0.805 ***	0.683 ***	-	
4. Depression	0.784 ***	−0.621 ***	−0.707 ***	-
M	43.57	50.38	48.89	5.24
SD	13.91	18.37	12.74	4.78
Skewness	0.44	0.05	−0.39	0.59
Kurtosis	−0.18	0.58	−0.17	−0.86

1. Loneliness; 2. structural social capital; 3. cognitive social capital; 4. depression. N = 334, *** *p* < 0.001.

**Table 3:** The moderating effect of structural social capital on the relationship between loneliness and depression (N = 334).

Variable	B	SE	t	LLCI	ULCI	*R* ^2^
Intercept	5.988	0.871	5.940 ***	3.390	6.748	0.651 ***
Loneliness	0.199	0.015	12.901 ***	0.169	0.229	
Structural social capital	−0.049	0.011	−4.485 ***	−0.071	−0.028	
Loneliness × structural social capital	−0.002	0.001	−3.350 ***	−0.003	−0.001	
Gender	−0.838	0.322	−2.604 **	−1.471	−0.205	
Age	0.19	0.213	0.088	−0.401	0.438	
Economic status	0.166	0.214	0.773	−0.256	0.587	
Presence of chronic disease	0.919	0.340	2.705 **	*0* *.251*	1.587	

N = 334, ** *p* < 0.01, *** *p* < 0.001.

**Table 4:** Simple regression for structural social capital.

Moderator		Effect	SE	*t*	LLCI	ULCI
Structural Social Capital	−1SD	0.230	0.016	14.638 ***	0.199	0.261
	M	0.199	0.015	12.901 ***	0.169	0.229
	+1SD	0.168	0.020	8.415 ***	0.129	0.207

N = 334, *** *p* < 0.001.

**Table 5:** The moderating effect of cognitive social capital on the relationship between loneliness and depression (N = 334).

Variable	B	SE	t	LLCI	ULCI	*R* ^2^
Intercept	4.722	0.868	5.442 ***	3.015	6.429	0.651 ***
Loneliness	0.201	0.019	10.530 ***	0.164	0.239	
Cognitive social capital	−0.066	0.022	−3.042 **	−0.108	−0.023	
Loneliness × cognitive social capital	−0.002	0.001	−2.763 **	−0.003	−0.001	
Gender	−0.673	0.333	−2.024 *	−1.328	−0.019	
Age	0.047	0.218	0.217	−0.381	0.476	
Economic status	0.179	0.221	0.813	−0.255	0.613	
Presence of chronic disease	0.625	0.346	2.671 **	0.244	1.606	

N = 334, * *p* < 0.05, ** *p* < 0.01, *** *p* < 0.001.

**Table 6:** Simple regression for cognitive social capital.

Moderator		Effect	SE	*t*	LLCI	ULCI
Cognitive Social Capital	−1SD	0.226	0.021	10.950 ***	0.186	0.267
	M	0.201	0.019	10.530 ***	0.164	0.239
	+1SD	0.176	0.022	8.158 ***	0.134	0.219

N = 334, *** *p* < 0.001.

## Data Availability

The datasets generated and/or analyzed during the current study are not publicly available due to privacy and ethical restrictions.
